# Human-centered design of an exercise intervention for adolescent cancer patients: findings from a patient involvement workshop to inform intervention development

**DOI:** 10.1186/s13063-026-09686-4

**Published:** 2026-04-06

**Authors:** Dania Schütze, Jennifer Engler, Sarah Erhard, Micah Walker, Mirjam Dieckelmann

**Affiliations:** 1 Goethe University Frankfurt, Institute of General Practice, Theodor-Stern-Kai 7, Frankfurt am Main, 60590 Germany; 2 Gesundheitsamt Frankfurt am Main (public health authority), Breite Gasse 28, Frankfurt am Main, 60313 Germany; 3 Goethe University Frankfurt, Department of Pediatrics, University Hospital, Theodor-Stern-Kai 7, Frankfurt am Main, 60590 Germany

**Keywords:** Exercise oncology, Patient involvement, Pediatric oncology, Adolescents, Human-centered design

## Abstract

**Background:**

Only few exercise trials have been conducted in the immediate time after a child’s first cancer diagnosis. The Exercise CC Trial was designed to study the effects of exercise during the first 8 weeks following the diagnosis. However, young patients have not been involved in designing an exercise intervention in this care-intensive period of time in which complying with regular exercise regimens may be particularly difficult. To understand factors facilitating compliance and to prevent drop-outs in this critical period, this study aims to engage adolescents with cancer in designing an exercise intervention by incorporating patient perspectives in research development.

**Methods:**

A workshop was conducted with adolescents who had previously participated in exercise therapy during their cancer treatment. This interactive session allowed participants to share their experiences and contribute to the intervention design prior to enrolling patients in the trial. Data was collected through a group discussion, which was recorded and transcribed for qualitative content analysis using MAXQDA20 software, ensuring a comprehensive understanding of participants’ views.

**Results:**

The workshop, attended by four patients aged 17 to 20, highlighted various aspects of exercise therapy. Participants valued the flexibility of choosing between various exercises and noted the psychological benefits of physical activity, which provided them with a constructive outlet and relief during treatment. They also appreciated the physical improvements and the motivational impact of monitoring progress. Importantly, the study revealed the need for personalized exercise regimes that consider individual physical conditions and preferences.

**Conclusions:**

We present an innovative approach in pediatric exercise oncology inviting the perspectives of adolescents and young adults to participate in research and shape the design of intervention details during the care-intensive phase immediately following a cancer diagnosis. We describe our “lessons learned” in giving adolescents a voice and share their knowledge and insights into their specific needs and preferences of exercise regimens in trials. This approach ensures that intervention details align with young patients’ expectations, which can be opposite to experts’ assumptions.

**Trial registration:**

DRKS-ID: DRKS00032259. Registered on 10.07.2024.

**Supplementary Information:**

The online version contains supplementary material available at 10.1186/s13063-026-09686-4.

## Background

Children and adolescents are rarely given a voice when it comes to research on their health. Even though a recent scoping review found that children with cancer were more frequently asked about their opinion compared to any other pediatric condition [1], only 9% of all studies involved children with regard to intervention development [1]. Our aim therefore was to engage adolescents [2] with cancer in designing an exercise intervention within the research context of pediatric exercise oncology.

These insights will inform the 8-week exercise randomized controlled trial (RCT) “Exercise early to reduce corticosteroid-induced side effects in childhood cancer” (EXERCISE CC) designed to investigate the effects of early exercise intervention on reducing corticosteroid-related side effects in recently diagnosed patients with leukemia or lymphoma.

Adolescents diagnosed with cancer not only experience the emotional and organizational burden of the diagnosis as well as the typical side effects of the therapy, but also have an increased risk of developing chronic diseases such as diabetes mellitus and obesity later in life [3, 4]. Initial corticosteroid therapy in particular can cause metabolic complications. These include a decrease in muscle mass and function, hyperglycemia, lethargy, irritability, or the development of sarcopenic obesity, which is characterized by increased body fat mass combined with reduced muscle function [5–7]. Thus, promotion of physical activity is critical [8, 9] and studies have shown the positive effects on diverse clinical and psychosocial outcomes [10–13]. Corticosteroid therapy can disrupt glucose metabolism and induce muscle protein loss. Exercise may mitigate these effects by activating insulin-independent glucose transporters [14, 15] and stimulating muscle protein synthesis via mTOR pathways [16]. Still, clinical data on the efficacy of early exercise interventions—initiated immediately upon diagnosis—are lacking. Existing pediatric trials demonstrate encouraging outcomes, including gains in strength and endurance alongside reduced fatigue [17–20].

A study with young adults aged 18–39 years investigated exercise preferences and found them to be highly individual and sometimes contradictory [21]. The YOUEX study highlighted the need for personalized, adaptable exercise interventions rather than one-size-fits-all programs since participants’ preferences varied regarding the degree of supervision and structure (valuing accountability and professional guidance versus flexible routines and autonomy). Contradictions emerged in attitudes toward intensity with some favoring high-intensity workouts for efficiency, whereas others avoided them due to symptom burden like fatigue. Social preferences also diverged: group settings were motivating for some but overwhelming for others. A systematic review synthesized evidence on barriers and facilitators to exercise in childhood cancer survivors and identified that fatigue, pain, fear of injury, lack of motivation, and societal misconceptions about the young persons’ capabilities were perceived as barriers to physical activity. Conversely, facilitators included social support through family and peers, tailored exercise programs, autonomy in activity choices, positive health beliefs, and integration of physical activity into daily routines as normalizing elements and facilitators to engage in exercise. The systematic review concluded that conclusive evidence was still missing, however [22]. Consequently, we aimed to learn about the experiences of patients who already received treatment at the clinic and participated in the standard exercise therapy program early after diagnosis. Standard exercise therapy followed German S2k guidelines and included an individually adapted, supervised exercise therapy for pediatric patients over 3 years of age including structured and unstructured training and active play for 10–30 min 2–5 times per week [5]. Before developing the intervention, we wanted to hear the voices of patients. We wanted to know what they found difficult, what they found helpful, what motivated them to exercise, and what exercise meant to them.

## Methods

### Design

Guided by human-centered design principles, we conducted a workshop with adolescents with the aim of incorporating their experiences and recommendations into the design of an exercise intervention [23–27].

### Recruitment and participants

For recruitment, we targeted adolescent patients^1^ aged 12 years or older [2] who had participated in exercise therapy during or after their active treatment at the University Hospital Frankfurt am Main in Germany. Recruitment was carried out via a poster displayed at the ambulatory center for oncological and hematological pediatric patients. The poster contained the invitation to actively participate in sports science research and provided contact information as well as two potential dates for the workshop. Additionally, exercise therapists and the psychosocial team approached potential candidates directly. Our inclusion criteria stipulated that candidates should have experience in exercise therapy, be post-treatment, and be capable of interacting in a group setting. Patients who were still undergoing acute treatment and therefore had to comply with strict hygiene and isolation rules were not addressed.

Participants were informed that the workshop was being conducted as part of a study and were provided with information on the use of data in accordance with the General Data Protection Regulation (GDPR). Participants then provided written informed consent. In the case of patients who were not of age, their parents also signed the consent form.

Participants each received a €25 voucher as compensation. Moreover, we provided a reference letter-styled certificate to acknowledge their initiative to participate in research.

### Setting and data collection

The workshop was designed by the author group, consisting of health scientists with experience in qualitative and participatory methods and sports therapists. It took place in April 2023 at the indoor exercise facilities of the Department of Pediatrics at the Frankfurt University Hospital and lasted 90 min. The session was hosted by two moderators (DS and SE), one female medical scientist—formerly unknown to the participants—and one female exercise therapist that some of the participants had already met during active treatment. The researchers and the participating adolescents sat together in a large semi-circle in front of a mirror wall. There were various seating options such as stools, benches, and mats and the adolescents were free to choose the seats that were most comfortable for them. The moderators led them through the workshop with a number of key questions (see Appendix A). The aim was to find out what “good days” and “bad days” felt like for the adolescents during exercise therapy at the clinic, whether and how they did sports at home during therapy, and whether they had any specific tips for conducting exercise therapy. In addition to the key questions, a natural exchange with spontaneous additional questions developed in the course of the conversation. The presence of the exercise therapist ensured that any uncertainties regarding sport-specific topics were addressed through targeted questioning, comprehensively illuminating all relevant aspects of the research question. During the discussion, one of the moderators wrote down statements and keywords on cards and stuck them on the mirror wall for everyone to see. The workshop was audio-recorded with the consent of the participants.

### Data analysis

Qualitative content analysis [28, 29] was based on the notes taken on cards during the workshop, the audio file, and a verbatim pseudonymized transcript of the audio recording. We analyzed the statements in terms of the requirements, recommendations, and suggestions that the adolescents gave us as experts with lived experience, with regard to the design of the exercise intervention. Three researchers (DS, JE, MD) used the notecards and the audio recording to develop inductive categories to structure the participants’ statements. Subsequently, one researcher (DS) used MAXQDA20 software to code the transcripts and form subcategories that filled these categories empirically [30]. In the following, these categories are presented with the respective experiences and perspectives of the participants (P).

## Results

Four patients (three males, one female) aged 17 to 20 participated. The group included one middle-aged adolescent and three late adolescents. All patients were treated in Goethe University Frankfurt, University Hospital, Department of Pediatrics, hematology and oncology for oncological disease (sarcoma, leukemia, brain tumor, lymphoma), one without remission. During treatment they all lived at home with their parents and inpatient stays alternated with days at home. The results of the workshop are presented below in the categories of perception of sport, impact of physical activity on well-being, and relevant aspects in the design of training sessions.

### What is perceived as “sports”?

Through the narrations of their time spent in exercise therapy, it became clear that a wide range of exercises and movements were perceived as “sport” by the participants. All levels of intensity and all main forms of motor stress were relevant for them. These included strength training, endurance training, ball sports, Zumba, and yoga, but also movements that require less strength, such as balance training, exercises for fine motor skills, stretching exercises, breathing exercises, relaxation, and meditation.

### Effect of physical activity—increased self-efficacy

During the conversation, it became evident that exercise therapy and movement helped the participants feel less powerless and experience a sense of self-efficacy. Exercise was “*just like a reason for me to get out of bed*” (P4). They described how it was beneficial to them and how they felt better afterwards, both physically and mentally:

It really does you good. And you feel a bit better too. Mentally. Because you think you’ve done something. [...] You’re lying in the hospital all the time. You’re in your bed. And you’re not really doing anything to beat the cancer. Other people then tell you: You’re doing a good job. And I think to myself: I’m not doing anything. I’m lying around. I have to put up with it. (P1)

Exercise offered a sense of normality and gave them a feeling of progress through regaining strength and energy that was diminished by their illness and treatment:

So bit by bit, workout after workout, I just realized once again: Oh my, my muscles are back. [...] Then when I looked at myself in the mirror, there was just a kind of a wow effect. (P1)

In this context, sore muscles were explicitly described as something positive by some participants, as “*a response from the body […] ‘yes, I did something’!*” (P3).

The idea that exercise helps in regaining strength and mobility for activities you look forward to after cancer therapy, such as walking a dog and practicing tricks with it, was described as an additional motivating factor. It reestablished an active, constructive role—setting realistic goals, practicing, and tracking gains.

One participant formulated his advice for other patients as follows:

You should definitely not lie there wallowing in your illness. That’s what I did in the beginning. I just said I’m sick now. I don’t have to do any sports. I can do all that afterwards. But that was a huge mistake for me, because I gained a bunch of weight as a result, and lost a lot of my muscle strength. [...] You should buck up and really try to rise above the circumstances despite your illness. (P3)

Another participant emphasized that he was happy to have something to do during his hospital stay. He enjoyed doing sports, especially on good days, as it helped him to clear his head and distract himself.

### Key components of exercise therapy design

All participants agreed that doing exercise during therapy was both important and had a positive effect. However, they outlined general conditions that made it easier to exercise. The following aspects thus acted as facilitating factors, while their absence or violation acted as barriers.

#### Having the opportunity to choose

In particular, the participants emphasized that it was helpful to be able to choose between different types of exercises:

What I liked a lot was that you could really exercise whatever you really wanted to. You weren’t forced to just cycle for 30 minutes or just do strength training. You could also say, ‘Nah, I don’t feel like that right now.’ Then we’d do something else, toss a ball around or something. (P2)

Some participants also pointed out the possibility of exercising not only in the sports room, but also in their own room. In this way, patients could exercise to the best of their ability, even if they were in poor general condition:

Definitely bring the sport to the person. Into their room. In my case, they also took the exercise mat with them so that we could do exercises on it, or they would bring the bike up to my place, for example, when I didn’t have the strength to go down. And I think that helps a lot. (P4)

#### Taking deficits seriously

In this context, the adolescents stressed that it was important for them that their deficits and complaints were taken into account and that the type and intensity of the exercises corresponded to their current general condition. One person even suggested always asking about current complaints at the start of an exercise session:

Something else that could help: If someone asks: What hurts? And what’s bothering you right now? As the first thing, right after greeting you. And then offer things that you can do that match your complaints. And also ask beforehand how much strength the person has. (P4)

For the participants, taking deficits seriously also meant that patients can demand the right to rest:

When I had such a bad day, I just didn’t get out of bed. If at all, then to go to the toilet, but sometimes I honestly thought to myself, I’ll just stay in bed and go to the toilet tomorrow. [...] I allowed myself the right to just stay in bed. (P3)

But there are days when things just don’t work. And there are also days when they do work and when it’s fun. (P1)

The group was in agreement that exercise therapy every 2 to 3 days with corresponding days off in between was appropriate and feasible.

#### Tracking training progress

Two participants pointed out the importance and motivational effect of monitoring progress by measuring specific parameters during training sessions:

We also tracked the things. My muscle measurements. And then you just felt any changes. […] So, if you don’t do that [tracking] [...] you don’t have any goals or any visible progress. So, that’s just important. (P1)

On the one hand, monitoring helps to make regained strength and performance visible, thus supporting self-efficacy. At the same time, however, it could lead to frustration if it shows deterioration:

At the same time, I also find it frustrating. Because, for example, if you have done less or just couldn’t. Then, for instance, you gained or lost a lot of weight and you no longer like yourself because of it. That is tracked all the time. It feels like you are a failure[...] But at the same time, it’s good. Because when you gain strength, you can see the progress, like he said. (P4)

#### Session atmosphere

The participants described that the therapist’s demeanor played an important role in making the exercise sessions more pleasant for them:

In any case, a charismatic appearance is a must. A smile [...] so that the patient feels comfortable etc. (P2)

One participant emphasized that they especially appreciated the conversations during the exercises that also distracted them in a positive way:

Well, for me it was always the conversations I had during the sports that I enjoyed the most. That way you don’t focus on the sport you’re doing, when you’ve lost your stamina or gotten weak [...] So, distractions during sport are very important, at least for me. (P3)

Music also helped to focus less on the exercises and also provided a pleasant atmosphere, especially when conversations could not fulfill this function. It motivated and “*always influences the body a little*” (P4).

#### Exercise at home

During the course of the therapy, the adolescents were repeatedly at home for several days at a time. They reported that they did more sport during their time in the clinic than at home. While they actively participated in the sports activities on offer during their inpatient stays, the young people tended to use the days off at home to rest:

So during the therapy, I didn’t do any sports at home at all. I said to myself, I’m not in the hospital now, so I don’t have to do sports. Hospital is actually just like school. You just do physical education, like everyone else. And at home, you’re just at home [...] So at home, I was just on the sofa at my room, watching my series or doing puzzles with my sister or something. (P3)

Some of them described going for a walk with friends from time to time and emphasized that they needed someone to encourage them to exercise as it is difficult to motivate yourself alone:

Some friend that visits me and says [...] ‘Well, you lazy thing, we’re going to do something now.’ That would have helped me. (P3)

When asked whether they could imagine using supporting materials such as videos at home, two participants said that they would find videos helpful, as well as handouts with exercises that might even be used as “homework”:

That you can get homework like that. Or print out sports exercises on a piece of paper. It doesn’t have to be sports exercises. It could be yoga, it could be balance training. And I think that would be really good. (P4)

And would you have liked to have had it checked, i.e. by us? (Exercise therapist)

Oh, it would be good if it were checked, because otherwise I won’t do it. (P4)

## Discussion

Participants described the positive effects that exercise had on them: they felt physically and mentally stronger, experienced self-efficacy, and appreciated the distraction that exercising provided. They emphasized the importance of tailoring the content of the sessions to their physical condition and preferences. These findings are consistent with the results of previous studies [31, 32]. The workshop provided us with insights for intervention development, for routine care, and also with regard to the methodological feasibility of involving adolescents in this context.

### Lessons for intervention development and routine care

The adolescents did not focus much on specific exercises, but mainly pointed out that individual framework conditions should apply to exercise therapy in terms of intensity, types of sport, main muscular strain, etc*.* However, it makes structured exercise intervention development difficult for an RCT, as the intervention details should be outlined [33].

Nevertheless, the workshop played a pivotal role in clarifying the framework of the EXERCISE CC intervention. To instill a sense of control and autonomy in patients during their workout routines, we plan to use visual exercise cards, complemented by a semi-preplanned framework for a multimodal training regimen. To enhance motivation, some extrinsic motivators should be reinforced during the intervention. A visual reward system with levels or the completion of a picture after completed training units is conceivable, perhaps in social (but anonymous) comparison. The workshop also helped us to determine the frequency of training for both the intervention and sham intervention groups. Moreover, we plan to include online training sessions for children and adolescent training at home as elements of the intervention. However, safety must be considered, and arrangements regarding insurance must be discussed before delivering exercise therapy at home. Exploration of factors in successful implementation is underway [34].

We have learned that some of the participating adolescents want exercise therapists to address their physical deficits and individual physical problems in therapy. This is also common practice in standard care. However, it is interesting to note that the efforts to work in a resource-oriented way (Where does it not hurt? What can the patient do without problems?), as recommended by the expert consensus, were not pivotal for the participants [5, 35]. We therefore want to design the intervention in such a way that patients have the opportunity (but do not have to) choose which physical problem they would like to work on in each session. In contrast, there does not necessarily need to be an over-focus by exercise therapists on sore muscles. For the participants, it was more of a natural physical feedback.

An important takeaway from the study is that monitoring body composition and assessing motor performance are not necessarily seen as a burden (e.g., stressing undesired bodily changes) but could be seen as a feedback aid. An important topic for future research could be the potential impact of visualizing physical activity levels, progress (and regression) in physical performance on patient motivation given the fluctuation in physical activity and the tendency of physical deconditioning associated with medical treatment regimens. Not only have visualization techniques been proposed for motivational purposes—a recent study has explored physicalization of activity levels as a feedback aid to help children tangibly understand their physical performance or activity [36].

With regard to the design of the sham intervention for the control group and the ethically relevant aspect of withholding therapy, the adolescents telling us that for them exercise therapy starts with walks and minimally intensive exercises is of great importance [37].

### Methodological reflection

Our experience shows that it is feasible and beneficial to involve adolescents with cancer in research. Meeting at the clinic’s gym facilitated recalling experiences related to this setting and gave the participants the opportunity to utilize exercise materials as we met. For example, they threw a ball to each other while talking, which further loosened up the atmosphere. The adolescents showed lots of engagement, as they stayed focused for 90 min. Afterwards, they told us that they enjoyed taking part in this workshop. Three participants left us their e-mail address to be kept up to date on the study.

It was very helpful to have an exercise therapist who knew some of the adolescents as one of the moderators. It is possible that participants are more reserved about criticism in such a setting. However, in the course of our workshop, it became clear how important it was for the participants and the moderator to be able to draw on shared memories and knowledge. The exercise therapist was able to ask specific questions and also refer specifically to individuals.

This one-time participatory workshop invited adolescents to share their perspective on exercise therapy during early anti-cancer treatment. In terms of patient involvement, our workshop operates at the consultation level [25, 38]. As a human-centered design approach, the workshop formed the basis for developing the intervention, with findings derived from qualitative analysis. Throughout the process, the researchers consistently adhered to a core principle of patient and public involvement by involving the adolescents as experts with lived experience and creating an atmosphere characterized by openness, empathy, and appreciative communication [38]. The objectives of the workshop were communicated transparently. However, transferring their knowledge into intervention modules was done by the researchers. If resources had allowed, it would have been helpful to engage adolescents further in the process of designing the intervention. Since the group wished to be informed about future project evolvement and enjoyed working together, a further hands-on workshop with direct feedback on the delivery of the different intervention modules or comments on videotaped intervention sessions would be a future step to fine-tune the intervention and provide more details.

### Limitations

The participants of this workshop were all inclined to engage in exercise therapy in a clinical setting and generally enjoyed physical activity. There may be patients who require different activities to motivate them, who perceive muscle soreness more negatively than others, or who feel more pressure during routine assessments. All of the participants were adolescents in the middle or late stages of development. It was not possible to incorporate the perspectives of younger children into this workshop. Still, such a workshop will never be able to cover all perspectives and does not aim to generate results that can be generalized to all patients. The adolescents have provided important insights into their experiences and thoughts so that the patient perspective can be taken into account in the development of the intervention.

## Conclusion

We gained insights into the meaning of being physically active from the perspective of adolescents in the healthcare-intensive period after diagnosis and learned about organizational factors that help with patient-centered intervention development during this time. Involving adolescents is possible in this context and knowing their perspective is not only beneficial for research but also important for routine care.

## Supplementary Information

Supplementary Material 1.

## Data Availability

The datasets generated and/or analyzed during the current study are not publicly available due to data protection reasons but are available from the corresponding author on reasonable request.

## Abbreviation

RCT: Randomized controlled trial

## Footnotes

1 We refer to the American Academy of Pediatrics’ definition of the stages of adolescence. According to this definition, early adolescence is defined as ages 10 to 13, middle adolescence as ages 14 to 17, and late adolescence as ages 18 to 21.
