# Supplementary material for: Human-centered design of an exercise intervention for adolescent cancer patients: findings from a patient involvement workshop to inform intervention development
Source: Trials. 2026 Apr 6;27:293. doi: 10.1186/s13063-026-09686-4 (PMC13067389; doi:10.1186/s13063-026-09686-4)
Supplement: Supplementary file 1 — Supplementary Material 1. [file 13063_2026_9686_MOESM1_ESM.docx]

**Guide**

| **Welcome and introduction** | |
| --- | --- |
| Welcome – it’s nice to see you all here. You've already received some information, but let me just briefly summarize again what we're going to talk about today: A **study on sports therapy for children and adolescents with cancer** is to be carried out here at the clinic and **we need your help for this.** You have **all been treated here** and have had **experiences** with sports therapy. We are interested in them because we want to **design** the **exercise program in the study in such a way that it is pleasant for other patients and they have the desire and strength to participate. You are the best people to tell us what helps and what doesn't**. So you can use your experience to help ensure that sports therapy can support patients even better in the future. Very important: There is **no right or wrong** here today. It's simply a matter of you telling us a bit about how you have fared with the exercise here.  Now you probably want to know **who we actually are**: Some of you already know [name exercise therapist]. She's a sports therapist here at the clinic. I'm [name medical scientist]. I'm a researcher here at the university and I'm helping to prepare the study on sports therapy.  As I just said, we simply want to talk to you about sports therapy and exercise. Basically, you can **just talk away**, so you don't have to ask for permission or anything. We don't have any special rules, except that smartphones are best left in your pocket and set to silence mode. We always write something on these **little cards** and stick them on the mirror and if you want, you can also write something on the cards yourself. In case we don't get what you are saying or miss something, we also **record** what is being said with this **recording device**. This is just to be on the safe side in case we need to listen to something again. Nobody will hear it except us. Do you agree to that?  Basically, of course, you only have to share with us what you want to share. There is no obligation to tell us anything.  Before we start, we think it would be great if you could also briefly introduce yourselves, because you don't know each other yet. Ideally, each of you should say your **name**, how **old** you are, when you were in **therapy** here and whether you're more of a **couch potato** or a **jock**, or something **in between**.  Do you have any questions before we start? Otherwise we'll start with the introductions. Who would like to start? *(start recording)* | |
| **Topic** | **Questions** |
| Open beginning | When you think back to your time here in the clinic and what it was like with sports and exercise: What advice would you **share with** children or adolescents who have just received their **first diagnosis** and are wondering **whether they should take part in sports or not**? |
| Sports therapy in the clinic ("good" days) | - What was a day like when you **felt like** doing sport and maybe it was even **easy** for you? - **What did you enjoy** about sports therapy - what did you particularly like?   - **Which sport, games or movements** did you enjoy the most? (If you like, you can also demonstrate)   - What sports or games or movements can you think of that can be really **strenuous but** were **helpful or fun**?     - Do you remember roughly how long you did them for?   - Did you sometimes get **sore muscles** during the therapy?     - How was it for you/you all? (if applicable, more specifically: Did it stop you from doing sport again?) - Approximately **how many times a week** did you take part in sports therapy here at the clinic? *(transition to the next block)* |
| Sports therapy in the clinic ("bad" days) | - What were the days when you **didn't take part**?   - **What helped you** to **move** on days when you weren't feeling well, or perhaps **to get out of bed** at all?   - **Which movements helped** or **worked** well on such days? (If you like, you can also demonstrate this.)   - Was there a particular time of day when it was easier for you to do sport?   - What was **not helpful** or made it **more difficult** for you?     - If applicable: And apart from the fact that you felt bad/weak on those days, were there any **other reasons or factors that made you feel less like** moving **or had less energy**? |
| Sport at home | - Did you do any **sport** when you were back **home**?   - **What** did you do/trained at home?     - How often? How long? With whom? - We are thinking about different ways of **giving** patients something **to do** at home, so they can also exercise there. For example, **training videos, a training plan** to check off or even small **sports sessions via video conference** with one of the sports therapists.   - What do you think? What would you like to do? What would help you most to exercise at home? |
| Intervention | We already said at the beginning that a **sports program** is to be developed **for a study**. And in such studies, **a few things** are **usually measured and examined at the beginning and end**, e.g. waist circumference, upper arm circumference, endurance, strength, balance.   - What do you think of such tests and measurements? - *Depending on the feedback:* Imagine these measurements and examinations are to be carried out in the study: Do you have any ideas about what would help to make this less unpleasant? - Who do you think should explain to patients what is being measured and examined? (The doctor? Or the person who then carries it out?) |
| Open closure | - Is there anything else you would like to say on the subject that you haven't yet gotten off your chest? |
| **Conclusion and evaluation** | |
| Thank you very much for giving us so much important information and sharing your experiences with us. We will now take another look at the answers and everything on these cards afterwards and then consider together with the sports therapists what a good exercise program for the study might look like. If you want, we can keep you up to date on the study and can also see whether you can get involved in the further course of the study if you feel like it, e.g. trying out exercises or telling medical students about your experiences. If you would like us to keep you up to date, please write your name and e-mail address here and we can send you an update by e-mail. | |
